# Viral metagenomic analysis of human bocavirus in pediatric pneumonia: detection pattern and genetic characterization

**DOI:** 10.3389/fcimb.2026.1868618

**Published:** 2026-07-27

**Authors:** Ju Zhang, Jiaheng Chen, Ming Hu, Jiaping Wang, Songyi Ning, Wen Zhang, Runfeng Sun

**Affiliations:** Department of Clinical Laboratory, Joint Institute of Pathogenic Biology, Donghai County People’s Hospital & School of Medicine of Jiangsu University, Lianyungang, Jiangsu, China

**Keywords:** bronchoalveolar lavage fluid, human bocavirus 1, lower respiratory tract infection, viral metagenomics, virome

## Abstract

**Background:**

Human bocavirus (HBoV) is frequently detected in pediatric respiratory samples, but its clinical role remains difficult to interpret because of asymptomatic shedding and frequent co-detection with other pathogens. Data from bronchoalveolar lavage fluid (BALF), which more directly reflects the lower respiratory tract, remain limited.

**Methods:**

This retrospective study analyzed 179 BALF samples collected from pneumonia patients in the Jiangnan region of China between July and December 2025. Metagenomic next-generation sequencing (mNGS) was used for HBoV detection, mNGS-derived abundance estimation, genotype assignment, and genome coverage analysis. VP1 and NS1 gene fragments were used for phylogenetic analysis, and recombination screening was performed using RDP4.

**Results:**

Using the predefined ≥10-read mNGS screening threshold, HBoV signals were detected in 22 of 30 pediatric samples (73.3%; 95% CI, 54.1–87.7%) and in none of the 149 adult samples (0%; 95% CI, 0–2.45%), showing an age-related detection pattern in this cohort (Fisher’s exact test, P = 6.91 × 10^-^²²). HBoV1 was assigned as the dominant genotype in all HBoV mNGS signal-positive samples. RPM values varied among these samples, but they should be interpreted as mNGS-derived relative abundance rather than absolute viral load. Genome coverage analysis and partial VP1/NS1 phylogenetic placement provided additional support for HBoV1 read-based detection and genotype assignment. RDP4 analysis did not detect recombination events involving the study-derived VP1 or NS1 fragments.

**Conclusions:**

HBoV1 was frequently detected in pediatric BALF samples in this retrospective cohort, suggesting that HBoV1 signals may be relevant to the interpretation of some pediatric lower respiratory tract samples. However, because qPCR validation, healthy controls, and a comprehensive multi-pathogen co-infection assessment were not included, these data do not establish HBoV1 as the direct causative agent of pneumonia. Larger studies with quantitative validation and more complete clinical data are needed.

## 1 Introduction

Respiratory tract infections are common among the general population and exhibit a high incidence rate in children (Shi et al., 2017). Clinical studies have demonstrated that various viruses can cause lower respiratory tract infections, including respiratory syncytial virus, influenza virus, and adenovirus (Jain et al., 2015). These viruses account for a significant proportion of pediatric hospitalizations and show specific seasonal fluctuations in prevalence (Obando-Pacheco et al., 2018). With the advancement of diagnostic technologies, several previously under-recognized viruses have been gradually incorporated into research priorities, with human bocavirus (HBoV) being one prominent example.

HBoV was first discovered in respiratory samples in 2005 and belongs to the genus *Bocaparvovirus* within the family *Parvoviridae* (Allander et al., 2005). Since then, numerous studies have detected this virus in populations across different countries and regions, finding that its detection rate is relatively high in pediatric respiratory samples (Song et al., 2010; Ahn et al., 2014). Some studies suggest that HBoV infection may be associated with lower respiratory tract diseases in children, and common clinical manifestations include cough, fever, and pneumonia (Zhang et al., 2008, Zhang et al., 2021). However, the pathogenic role of HBoV remains somewhat controversial. On one hand, some researchers have detected HBoV in asymptomatic individuals, suggesting that the virus may exhibit persistent infection or asymptomatic shedding (Jartti et al., 2012). On the other hand, HBoV frequently co-exists with other pathogens in respiratory samples, which makes it difficult to clarify its independent role in disease (Tan et al., 2025). Therefore, a systematic analysis of HBoV in real-world clinical samples remains necessary.

From a molecular perspective, HBoV is a single-stranded DNA virus with a relatively small genome, yet it encodes essential structural and non-structural proteins (Schildgen et al., 2012). Existing studies generally classify HBoV into four major genotypes (HBoV1–4) based on genetic sequence variations (Guido et al., 2016). Certain differences exist in the tissue distribution among these different genotypes. Previous research has widely established that HBoV1 is more commonly found in respiratory samples and is closely associated with respiratory tract infections (Shao et al., 2021). In contrast, HBoV2, HBoV3, and HBoV4 are more frequently detected in gastrointestinal samples (Nora-Krukle et al., 2018). This variation in distribution suggests that different genotypes may possess distinct biological characteristics and infection sites.

In recent years, metagenomic next-generation sequencing (mNGS) has been increasingly used for lower respiratory tract infection diagnosis, including BALF-based pathogen detection (Li et al., 2022). This method does not rely on specific primers, allowing for the simultaneous detection of multiple viruses and the discovery of potential novel or low-abundance pathogens (Diao et al., 2022, Diao et al., 2023). This unbiased approach has become increasingly important in clinical infectious disease diagnosis (Chiu and Miller, 2019). However, systematic research on HBoV in bronchoalveolar lavage fluid (BALF) samples remains relatively limited. In particular, few studies have conducted a comprehensive analysis that integrates detection patterns, mNGS-derived abundance, and genetic features (Takeuchi et al., 2019; Wei et al., 2023).

Most previous studies on HBoV have focused on upper respiratory tract specimens, such as nasopharyngeal swabs or aspirates. Although these samples are useful for screening, they do not always reflect viral signals in the lower respiratory tract. Recent studies have supported the clinical utility of BALF mNGS in lower respiratory tract infections (Zheng et al., 2024), but HBoV-related BALF mNGS data remain limited. In addition, because HBoV is often detected together with other pathogens and may persist after acute infection, studies that combine detection frequency, relative abundance, genome coverage, and genotype-level characterization are still needed to better interpret HBoV signals in clinical BALF samples.

Based on this background, this study retrospectively investigated the detection pattern of HBoV in 179 BALF samples from pneumonia patients in the Jiangnan region of China during the second half of 2025. Our objective was to compare HBoV detection between pediatric and adult groups, describe mNGS-derived HBoV abundance in positive samples, and characterize the genetic features of HBoV1 based on genome coverage and VP1/NS1 phylogenetic analysis. The results of this study aim to provide regional metagenomic data for understanding HBoV1 in pediatric lower respiratory tract infections.

## 2 Methods

### 2.1 Study design, sample collection, and pre-processing

This retrospective observational study was based on residual BALF samples collected from patients with clinically diagnosed pneumonia. From July to December 2025, BALF samples were obtained from Yixing People’s Hospital and the Affiliated Hospital of Jiangnan University. The sampling frame included ICU pneumonia patients who required oxygen support, underwent bronchoscopy for clinical diagnostic purposes, and had sufficient residual BALF available for viral metagenomic sequencing.

Patients were included if they had a clinical diagnosis of pneumonia based on clinical symptoms, radiological findings, and routine clinical assessment. Samples were included only when the residual BALF volume was sufficient and the sequencing data passed quality control. Samples were excluded if the remaining volume was insufficient, the sample quality was poor, basic clinical information was incomplete, sequencing quality control failed, or repeated BALF samples from the same patient were identified.

Because this study was based on available retrospective clinical specimens, no formal *a priori* sample size calculation was performed. All eligible BALF samples collected during the predefined study period were included. A total of 179 BALF samples were analyzed, including 30 pediatric samples and 149 adult samples. Patients younger than 18 years were assigned to the pediatric group, whereas those aged 18 years or older were assigned to the adult group. Available demographic, clinical, and sequencing-related information is summarized in **Supplementary Table 1**, including age, sex, clinical diagnosis, sampling location, patient group, ICU admission status, oxygen requirement, hospitalization information where available, raw reads, clean reads, host-depleted reads, total HBoV-mapped reads, and genotype-level HBoV read counts.

Immediately after collection, BALF samples were placed in sterile centrifuge tubes and transported on dry ice to the Biosafety Level 2 laboratory at the School of Medicine, Jiangsu University. Frozen BALF samples were thawed on ice, and 600 μL of each original sample was transferred to a new centrifuge tube. The samples were vortexed for 5 minutes to ensure homogenization and then centrifuged at 15,000 × g at 4 °C for 10 minutes to remove cellular debris and large particulates. The supernatants were stored at -80 °C for subsequent viral particle enrichment, filtration, and nucleic acid extraction.

### 2.2 Library construction and sequencing

To enrich viral particles and reduce interference from free host-derived nucleic acids, 500 μL of the pre-processed sample supernatant was centrifuged at 12,000 × g for 5 min at 4 °C. The supernatant was then filtered through a 0.45 μm pore-size membrane to remove cellular debris and large particulates.

For nuclease treatment, 166.5 μL of the filtrate was mixed with 20 μL of 10× TURBO DNase buffer, 7 μL of TURBO DNase (Invitrogen/Thermo Fisher Scientific, Cat. No. AM2238, 2 U/μL; 14 U per reaction), 3 μL of Baseline-ZERO DNase (LGC Biosearch Technologies), 3 μL of Benzonase nuclease (Merck/EMD Millipore, Cat. No. 70664), and 0.5 μL of RNase A, DNase- and protease-free (Thermo Scientific, Cat. No. EN0531, 10 mg/mL; final concentration, 25 μg/mL). The final reaction volume was 200 μL. The mixture was incubated at 37 °C for 30 min to degrade free nucleic acids not protected by viral particles. DNase stop solution was not used before nucleic acid extraction (Kohl et al., 2015; Lewandowska et al., 2017).

Following enzymatic treatment, viral DNA and RNA were extracted using the QIAamp MinElute Virus Spin Kit (QIAGEN, Hilden, Germany; Ref. 57704) according to the manufacturer’s instructions. The extracted nucleic acids were used to synthesize first-strand cDNA using SuperScript III Reverse Transcriptase (Life Technologies/Invitrogen, Carlsbad, CA, USA; Cat. No. 18080-085) and 100 pmol of random hexamer primers. The second strand was synthesized using DNA Polymerase I Klenow Fragment (Vazyme Biotech, Nanjing, China; Cat. No. N104-01) to obtain double-stranded DNA. The resulting double-stranded DNA was used for library construction with the TruePrep DNA Library Prep Kit V2 for Illumina (Vazyme Biotech, Nanjing, China; Cat. No. TD503) (Li et al., 2015). A total of 179 sequencing libraries were constructed in this study. High-throughput sequencing was performed on the Illumina NovaSeq 6000 platform using a paired-end 150 bp strategy and dual-indexing for data acquisition and sample demultiplexing.

### 2.3 Bioinformatics analysis

The raw sequencing data were first processed using Illumina software for demultiplexing and paired-end sequence matching. All bioinformatics pipelines were performed on a Linux server cluster equipped with 256-core CPUs. Trim Galore v0.6.11 (Krueger et al., 2023) was used for quality control of raw reads, including adapter removal and trimming of low-quality bases with the parameters --phred33 --length 50 --stringency 3 --paired. Sequencing quality metrics were summarized at the sample level using FastQC v0.12.1 after trimming. Raw reads, clean reads after trimming, and host-depleted reads were reported as the total number of reads from R1 and R2 combined. Clean read percentage was calculated as clean reads after trimming divided by raw reads. Host-depleted percentage was calculated as host-depleted reads divided by clean reads after trimming. The FastQC-estimated sequence duplication rate was calculated as the mean percent duplicates of R1 and R2 after trimming (**Supplementary Table 1**).

The quality-controlled reads were aligned to the human reference genome (GCF_000001405.40) using Bowtie2 v2.5.0 (Langmead and Salzberg, 2012) and reads matching host sequences were removed to obtain host-depleted reads. Host-depleted reads were then used for both assembly-based and read-level viral screening. For assembly-based screening, MEGAHIT v1.2.9 (Li et al., 2016) was used for *de novo* assembly with the parameter --min-contig-len 200. Assembled contigs were searched using DIAMOND v2.1.18 (Buchfink et al., 2015) blastx against a locally constructed NCBI RefSeq viral protein database and were then imported into MEGAN v7.1.1 (Huson et al., 2007) for LCA-based taxonomic assignment. The RefSeq viral protein database was downloaded from the NCBI RefSeq viral FTP directory and locally constructed in September 2025, with protein accession identifiers retained in the FASTA headers.

To reduce the chance of missing low-abundance viruses that did not assemble into contigs, one read end of the host-depleted paired-end reads was also analyzed by read-level DIAMOND/MEGAN screening. For this step, reads were first searched against a viral subset of the NCBI nr protein database corresponding to the NCBI taxonomy group Viruses (taxid: 10239), which was locally constructed in July 2025. Candidate viral hits were then checked against the complete NCBI nr protein database constructed in the same period to reduce possible misclassification caused by conserved or non-viral homologous protein sequences. Potential viral sequences were screened using an E-value threshold of < 1 × 10^-5^. The DAA files generated by DIAMOND were imported into MEGAN for LCA-based taxonomic assignment.

HBoV detection and genotype-level read distribution were further evaluated using a reference database containing representative strains of HBoV1 to HBoV4 (HBoV1: DQ000495.1; HBoV2: FJ170279.1; HBoV3: GQ867667.1; HBoV4: FJ973561.2). Host-depleted reads were re-mapped to this database using Bowtie2 with the --very-sensitive-local setting and reads aligned to each reference genotype were counted using Samtools v1.13 (Danecek et al., 2021). Samples with a total HBoV-mapped read count of ≥10 were defined as HBoV mNGS signal-positive for screening purposes. This threshold was used to avoid interpreting single-read or very-low-read hits as positive, but it was not intended to represent a clinically validated diagnostic cutoff or a quantitative viral-load threshold. A threshold sensitivity analysis was performed using alternative total HBoV-mapped read thresholds.

Samples meeting the screening threshold were interpreted together with dominant genotype assignment, genome coverage breadth and depth, and VP1/NS1 phylogenetic evidence when sufficient sequence information was available. Because HBoV genotypes share conserved genomic regions, low-level reads mapped to non-dominant genotype references were interpreted cautiously and were not considered sufficient evidence of mixed-genotype infection. Genotype assignment was therefore based on concordant evidence from the dominant remapping signal, HBoV1 genome coverage patterns, and BLASTn or phylogenetic evidence from recovered HBoV-related VP1/NS1 fragments when available.

HBoV abundance was normalized using the reads per million metric to reduce the impact of differences in sequencing depth between samples. Because qPCR validation was not performed in this study, RPM was interpreted as an mNGS-derived relative abundance rather than an absolute viral copy number. For HBoV1 mNGS signal-positive samples, Samtools depth was used to calculate per-site sequencing depth across the HBoV1 reference genome (DQ000495.1), including coverage breadth, mean depth, median depth, and maximum depth (**Supplementary Figure 1**; **Supplementary Table 2**).

Three laboratory environmental surface control libraries were also analyzed to assess possible background viral signals from the tested laboratory surfaces. These controls included a PCR instrument surface control, a centrifuge inner-surface control, and a biosafety cabinet inner-surface control. The control libraries were processed using the same read-level DIAMOND/MEGAN screening workflow and targeted HBoV1–4 remapping procedure as the BALF samples. The controls were used as laboratory environmental background references and were not used for computational subtraction of taxa from BALF samples.

Read-level viral assignments were also used to summarize viral co-detection in pediatric BALF samples. HBoV was evaluated separately by targeted HBoV1–4 remapping. Other selected human- or vertebrate-associated viral genera were summarized from read-level DIAMOND/MEGAN assignments at the genus level. Bacteriophage-related taxa and viral groups mainly associated with environmental, algal, insect, giant DNA virus, retrovirus-like, or low-confidence background signals were not included in the co-detection heatmap to avoid overinterpretation.

### 2.4 Contig-guided recovery of partial VP1/NS1 fragments

Because the *de novo* assembled HBoV-related contigs did not provide complete HBoV genomes, we used a contig-guided local recovery strategy to obtain partial VP1 and NS1 fragments for genotype confirmation and phylogenetic placement. HBoV-related contigs generated by MEGAHIT were first checked by BLASTn (Altschul et al., 1990) against the NCBI nucleotide database. Contigs assigned to human bocavirus VP1 or NS1 regions were retained as VP1/NS1-related contigs for downstream local read mapping.

The retained VP1/NS1-related contigs were used as local references in Geneious Prime v2024.0.5 (www.geneious.com). Host-depleted paired-end reads from HBoV mNGS signal-positive samples were mapped back to these contigs using the low-sensitivity/fastest setting. The resulting local assemblies were visually inspected in Geneious Prime. Regions were retained only when they were supported by mapped reads, overlapped the corresponding VP1/NS1-related contig, and showed a consistent HBoV-related BLASTn assignment. Poorly aligned terminal regions and regions without local read support were not used for downstream phylogenetic analysis.

This procedure was designed to recover local HBoV1-related fragments rather than to reconstruct complete viral genomes or perform site-level variant calling. Therefore, we did not apply a fixed genome-wide minimum depth threshold or consensus-frequency threshold. The recovered sequences were treated as study-derived partial VP1 or NS1 fragments and were used only for genotype-level interpretation and phylogenetic placement. They were not used for whole-genome analysis or site-level nucleotide variation analysis.

The obtained partial VP1 and NS1 fragments were aligned with representative HBoV reference sequences using MAFFT v7.526 with the --auto option (Katoh and Standley, 2013). The alignments were trimmed using TrimAl v1.5.0 (Capella-Gutiérrez et al., 2009) with the --automated1 option to reduce poorly aligned positions. Maximum likelihood phylogenetic trees were constructed using IQ-TREE v2.3.6, and ModelFinder was used to select the best-fit substitution model with the -m MFP option. Ultrafast bootstrap analysis with 1,000 replicates was performed using the -bb 1000 option (Minh et al., 2020).

Recombination screening was performed using RDP4 v4.101 (Martin et al., 2015) based on the VP1 and NS1 nucleotide alignments. The RDP, GENECONV, BootScan, MaxChi, Chimaera, SiScan, and 3Seq methods were used, with Bonferroni correction and a highest acceptable P value of 0.05. The sequences were treated as linear DNA sequences. Because the available sequences were partial fragments rather than complete genomes or full-length genes, the phylogenetic and recombination analyses were interpreted only within the analyzed regions. The RDP4 screening results are summarized in **Supplementary Table 3**. The final phylogenetic trees and viral domain diagrams were visualized and refined using ChiPlot (Xie et al., 2023)(https://www.chiplot.online/) and Adobe Illustrator 2025 v29.0.

### 2.5 Statistical analysis and visualization

All statistical analyses and figure plotting were performed using R software v4.3.1 (Ihaka and Gentleman, 1996). Data processing was primarily conducted using the tidyverse v.2.0.0 suite of packages. HBoV mNGS signal-positive frequencies between pediatric and adult groups were compared using Fisher’s exact test. Exact binomial 95% confidence intervals were calculated for detection frequencies. Because no HBoV mNGS signal-positive sample was observed in the adult group, the odds ratio and 95% confidence interval were estimated using the Haldane–Anscombe correction for effect-size reporting. Fisher’s exact test was performed using the original uncorrected 2 × 2 table.

For the threshold sensitivity analysis, Fisher’s exact test was repeated across alternative total HBoV-mapped read thresholds. No multiple comparison adjustment was applied because the main analysis included a single predefined group comparison, and the threshold sensitivity analysis was interpreted descriptively rather than as a set of independent hypothesis tests. Differences in mNGS-derived HBoV abundance between groups were evaluated using the Wilcoxon rank-sum test because RPM values were not normally distributed. Log10 or pseudo-log transformation was used for visualization of RPM values.

For HBoV1 genome coverage heatmap visualization, per-site depth values were summarized into non-overlapping 50-bp genomic bins by calculating the mean depth within each bin. The binned depth values were then transformed using log10(depth + 1), and heatmaps were generated using pheatmap to display read distribution across the HBoV1 reference genome. For laboratory environmental controls and viral co-detection analysis, MEGAN normalized values were transformed using log10(value + 1) for heatmap visualization. Visualization results were produced mainly using ggplot2 v.4.0.1, ggpubr v.0.6.2, patchwork v.1.3.2, and pheatmap v.1.0.13.

### 2.6 Data availability

The raw sequencing data generated in this study have been deposited in the Genome Sequence Archive (GSA) of the National Genomics Data Center (NGDC), China National Center for Bioinformation (CNCB)/Beijing Institute of Genomics, Chinese Academy of Sciences, under BioProject accession PRJCA061470 and GSA accessions CRA040926 and CRA045159 (https://ngdc.cncb.ac.cn/gsa) (Chen et al., 2021). The HBoV1 VP1 and NS1 fragments have been deposited in GenBase under accession numbers C_AA360814.1 to C_AA360832.1 and are publicly accessible at https://ngdc.cncb.ac.cn/genbase.

## 3 Results

### 3.1 Detection and mNGS-derived relative abundance of HBoV in clinical samples

Across the 179 BALF libraries, the median number of raw reads per sample was 29,195,520 (IQR, 14,592,383–38,413,059), the median number of clean reads after trimming was 25,336,260 (IQR, 13,290,733–34,918,862), and the median number of host-depleted reads was 15,391,190 (IQR, 8,994,195–24,541,064). The median FastQC-estimated sequence duplication rate was 45.30% (IQR, 21.98–64.03%). Detailed sample-level sequencing metrics are provided in **Supplementary Table 1**.

**Figure 1:**
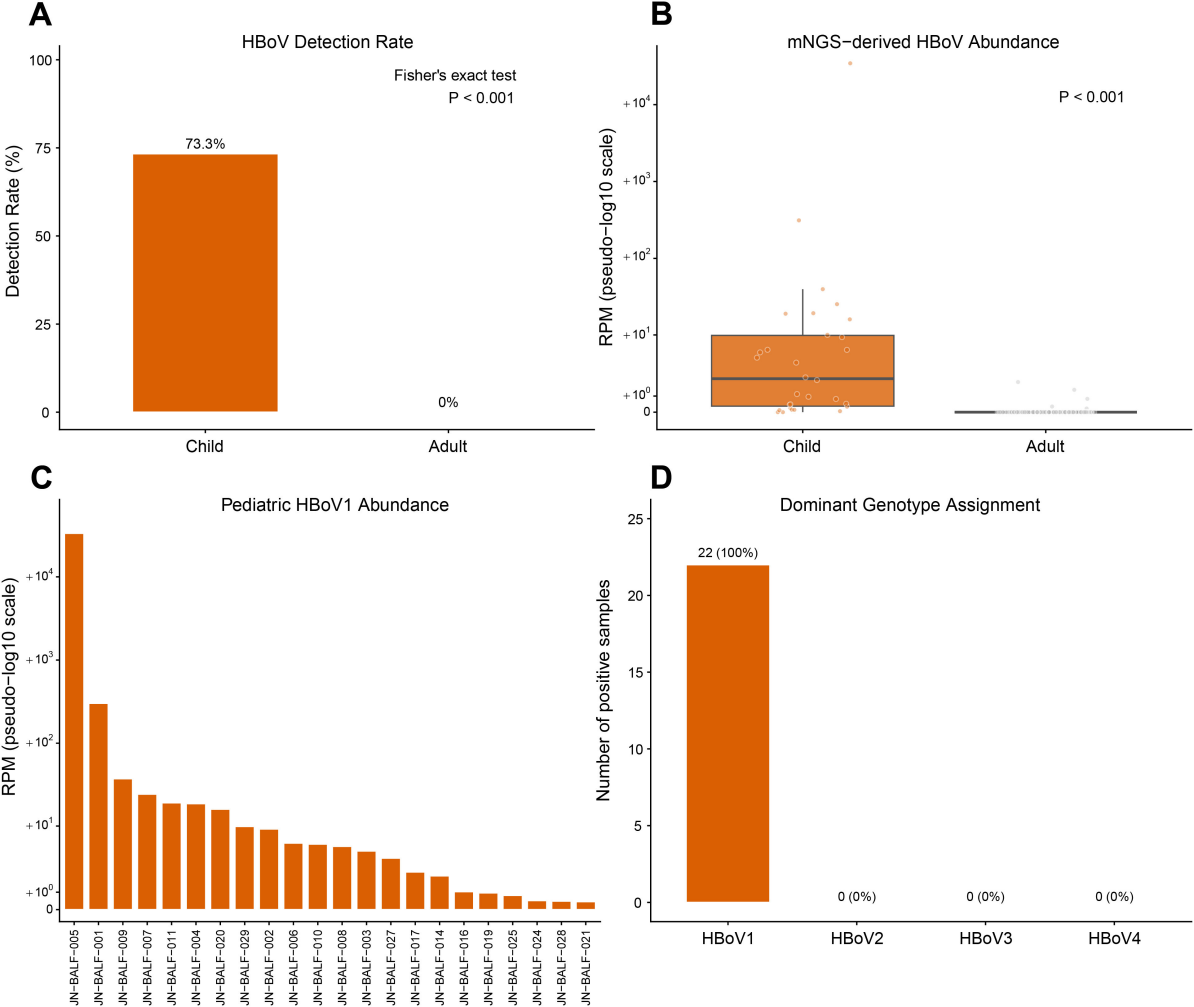
Detection, mNGS-derived abundance, and dominant genotype assignment of HBoV in clinical BALF samples. **(A)** Comparison of HBoV mNGS signal detection frequency between the pediatric group (n = 30) and the adult group (n = 149). A sample was considered HBoV mNGS signal-positive for screening purposes when the total HBoV-mapped read count was ≥10. Error bars indicate exact binomial 95% confidence intervals. Detection frequencies were compared using Fisher’s exact test. **(B)** Comparison of mNGS-derived HBoV abundance between the pediatric and adult groups. HBoV abundance was normalized using reads per million (RPM) and displayed on a pseudo-log10 scale. Differences between groups were evaluated using the Wilcoxon rank-sum test. **(C)** Distribution of HBoV1 abundance among HBoV mNGS signal-positive pediatric samples. Samples are sorted by RPM values from high to low. **(D)** Dominant genotype assignment among HBoV mNGS signal-positive pediatric samples. Genotype assignment was based on the dominant genotype-level read signal among HBoV1–4, together with genome coverage and VP1/NS1 phylogenetic evidence. Minor low-level mapping to non-HBoV1 references was not interpreted as mixed-genotype infection.

Based on metagenomic sequencing data from 179 BALF samples, this study analyzed the detection pattern of HBoV mNGS signals. Using the predefined screening threshold of ≥10 total HBoV-mapped reads, HBoV mNGS signals were detected in 22 of 30 pediatric BALF samples (73.3%; 95% CI, 54.1–87.7%) and in none of the 149 adult BALF samples (0%; 95% CI, 0–2.45%) (**Figure 1A**). Fisher’s exact test showed a significant difference between the pediatric and adult groups (P = 6.91 × 10^-^²²). Because the adult group contained zero signal-positive samples, the effect size was estimated using the Haldane–Anscombe correction, giving an odds ratio of 791.5 (95% CI, 44.1–14190.7). These results indicate an enrichment of HBoV mNGS signals in pediatric BALF samples in this cohort, although the limited number of pediatric samples should be considered when interpreting the detection frequency.

To examine whether this pattern depended on the selected read-count threshold, we performed a threshold sensitivity analysis using alternative total HBoV-mapped read thresholds. The number of signal-positive pediatric samples decreased as the threshold became more stringent, but the pediatric enrichment pattern remained evident at thresholds of ≥20, ≥50, ≥100, and ≥150 reads, whereas no adult sample met these thresholds (**Supplementary Table 4**). This analysis suggests that the age-related pattern was not driven only by the ≥10-read screening threshold, although low-read samples should still be interpreted cautiously.

Analysis of HBoV RPM values showed clear differences among signal-positive pediatric samples (**Figure 1B**). Notably, sample JN-BALF-005 showed the highest HBoV RPM value (RPM > 10^4^), while the RPM values of most signal-positive samples were concentrated within the range of 10^0^–10² (**Figure 1C**), reflecting heterogeneity in sequencing-based HBoV signal intensity among individuals. Genotype assignment showed that all HBoV mNGS signal-positive pediatric samples were dominated by HBoV1 (**Figure 1D**). Minor low-level mapping signals to non-HBoV1 references, when present, were interpreted cautiously and were not considered sufficient evidence of mixed-genotype infection, given the conserved regions shared among HBoV genotypes. Further HBoV1 reference-genome coverage analysis showed that HBoV1 reads were broadly distributed across the reference genome (DQ000495.1), rather than being restricted to a single short region. Detailed HBoV1 coverage metrics, including covered genome length, coverage breadth, mean depth, median depth, and maximum depth for each HBoV-positive pediatric sample, are provided in **Supplementary Table 2**. This broad coverage pattern provided supportive read-level evidence for HBoV1 presence and was consistent with the HBoV1 genotype assignment. However, genome coverage alone cannot exclude laboratory contamination, index hopping, or sample cross-contamination.

Laboratory environmental surface control libraries were further examined to assess possible background viral signals from the tested laboratory surfaces. Read-level DIAMOND/MEGAN screening showed that the viral signals in these controls were mainly phage-related or environmental viral taxa, including *Autographiviridae*, *Chaseviridae*, *Drexlerviridae*, *Inoviridae*, *Microviridae*, *Peduoviridae*, *Picobirnaviridae*, and *Schitoviridae* (**Supplementary Figure 2**; **Supplementary Table 5**). Targeted remapping against the HBoV1–4 reference sequences detected no HBoV1 reads in any of the three controls. Only two HBoV3-mapped reads were observed in the centrifuge inner-surface control, which was below the predefined screening threshold and was not accompanied by HBoV1 signal (**Supplementary Table 5**). These results did not support HBoV1 contamination from the tested laboratory environmental surfaces.

To evaluate viral co-detection, we further reviewed read-level DIAMOND/MEGAN viral assignments in pediatric BALF samples. Selected human- or vertebrate-associated viral genera were detected in a subset of pediatric BALF samples and are shown in **Supplementary Figure 3**. HBoV-related taxa were not included in this heatmap because HBoV was evaluated separately by targeted HBoV1–4 remapping. This analysis indicates that HBoV1 signals may occur either alone or together with other viral signals in some pediatric BALF samples, supporting a cautious interpretation of HBoV1 detection.

### 3.2 Phylogenetic analysis of HBoV1 VP1 and NS1 fragments

**Figure 2:**
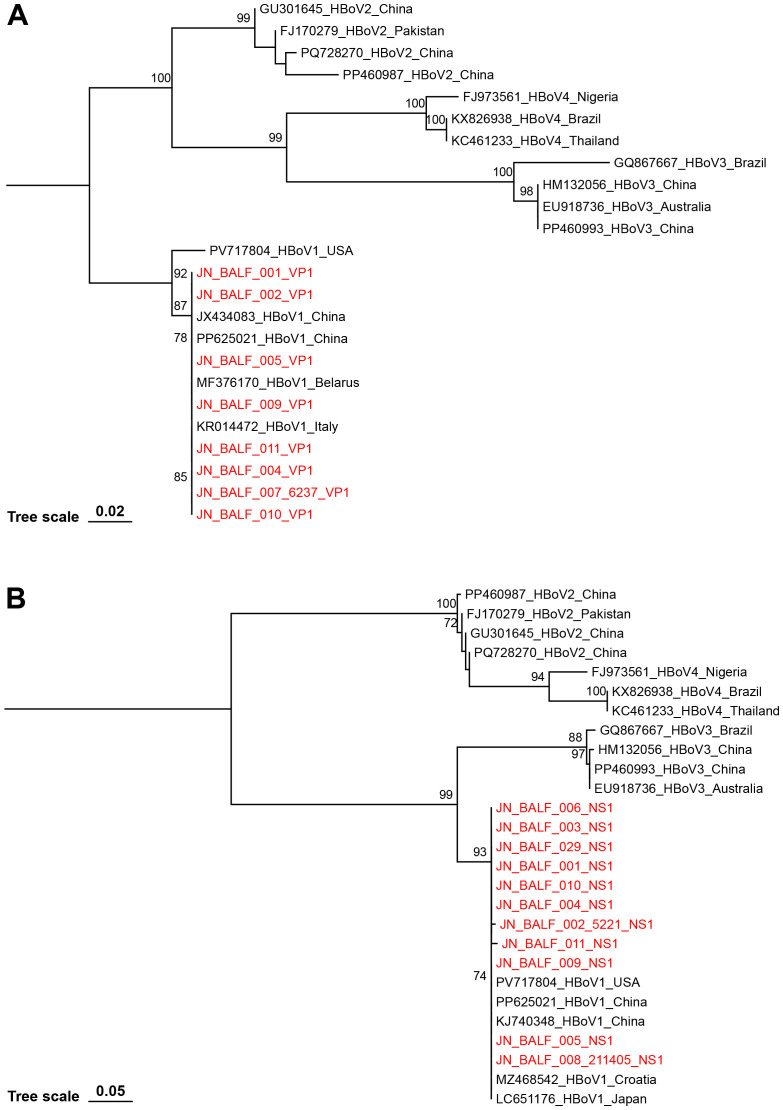
Maximum likelihood phylogenetic trees of HBoV1 constructed based on partial VP1 and NS1 fragments. **(A)** Phylogenetic tree constructed based on partial VP1 nucleotide fragments. **(B)** Phylogenetic tree constructed based on partial NS1 nucleotide fragments. Study-derived fragments are highlighted in red font.

To further evaluate the genotype assignment of HBoV, we constructed phylogenetic trees based on recovered partial VP1 and NS1 fragments. A total of 19 study-derived partial VP1/NS1 fragment sequences met the inclusion criteria and were used for phylogenetic analysis. In the VP1 phylogenetic analysis, the study-derived fragments clustered within the HBoV1 clade together with representative HBoV1 reference sequences (**Figure 2A**). Most fragments grouped closely with HBoV1 reference strains from China and other countries, supported by high bootstrap values, suggesting close relatedness within the analyzed VP1 region. In the NS1 phylogenetic tree, the study-derived fragments also clustered within the HBoV1 clade (**Figure 2B**). Together with the dominant HBoV1 remapping signal and HBoV1 coverage pattern, these independently evaluated VP1 and NS1 fragments supported the HBoV1 genotype assignment within the analyzed regions.

RDP4 analysis did not detect evidence of recombination in the VP1 alignment. In the NS1 alignment, one candidate recombination event was detected among HBoV3/HBoV4 reference sequences, supported only by GENECONV, but no recombination event involving the sequences obtained in this study was identified. Because the available VP1 and NS1 sequences were partial fragments rather than complete genomes or full-length genes, the phylogenetic and recombination results were interpreted only within the analyzed regions.

## 4 Discussion

This study characterized HBoV signals in BALF samples from a retrospective pneumonia cohort using viral metagenomic sequencing. HBoV mNGS signals were detected mainly in pediatric BALF samples, whereas no HBoV mNGS signal-positive samples were identified among the adult samples analyzed. All HBoV mNGS signal-positive samples were dominated by HBoV1, which is consistent with previous reports showing that HBoV is more commonly detected in infants and young children with respiratory tract infections and that HBoV1 is the major genotype associated with respiratory samples (Falahi et al., 2020; Petrarca et al., 2020; Trapani et al., 2023; Tan et al., 2025). The absence of HBoV signals in adults should not be interpreted as evidence that adults are not susceptible to HBoV infection. It more likely reflects the sample composition, previous exposure history, and detection pattern of this selected retrospective cohort (Ghietto et al., 2015; Jiang et al., 2016).

The relatively high HBoV mNGS signal frequency in the pediatric group should be interpreted in relation to patient selection and sample source. The pediatric samples were not collected from a general pediatric respiratory cohort. They were obtained from ICU pneumonia patients who required oxygen support and underwent bronchoscopy for clinical diagnostic purposes. This clinical setting may have enriched the cohort for more complex lower respiratory tract infections. BALF sampling may also capture lower-respiratory-tract viral signals more directly than upper respiratory tract specimens, although BALF collection is invasive and is usually performed only when clinically indicated (Diao et al., 2023; Wei et al., 2023). Therefore, the 73.3% value should be viewed as the HBoV mNGS signal frequency in this selected BALF cohort, rather than as a general prevalence estimate for pediatric pneumonia. When placed in a regional context, our findings are broadly consistent with previous studies showing that HBoV circulates among children with respiratory tract infections in China and neighboring Asian countries, and that HBoV1 is the major genotype detected in respiratory samples (Fry et al., 2007; Song et al., 2010; Ahn et al., 2014; Wen et al., 2025).

HBoV detection alone cannot establish pathogenicity. HBoV may persist after acute infection, and co-detection with other respiratory pathogens is common (Jartti et al., 2012; Tan et al., 2025). In the present study, the read-level viral co-detection analysis showed additional selected human- or vertebrate-associated viral signals in some pediatric BALF samples. This finding supports a cautious interpretation of HBoV1 detection, because HBoV1 signals may occur either alone or together with other viral signals. The available data therefore indicate viral detection and a cohort-level association, but they do not prove that HBoV1 was the direct causative agent of pneumonia. A more complete clinical and multi-pathogen assessment would be needed to distinguish active infection, co-detection, prolonged shedding, and incidental viral presence (Calvo et al., 2016; Sun et al., 2019; Tan et al., 2025).

The mNGS-derived HBoV signal intensity varied among HBoV mNGS signal-positive pediatric samples. Sample JN-BALF-005 showed the highest HBoV RPM value and was clinically diagnosed with bocavirus pneumonia, indicating a stronger sequencing-based HBoV signal in this case. However, this finding alone is not sufficient to establish causality or a direct association with disease severity. Previous studies have reported that high HBoV1 loads measured by quantitative assays may be associated with acute respiratory disease, whereas low-level detection or co-detection may reflect prolonged shedding, persistence, or a non-primary pathogenic role (Calvo et al., 2016; Jiang et al., 2016; Sun et al., 2019; Mijač et al., 2024). In this study, qPCR validation was not available, and RPM values were affected by sequencing depth, host-depletion efficiency, and library composition. The RPM values should therefore be interpreted as mNGS-derived relative abundance estimates rather than absolute viral copy numbers. Future studies with reserved sample aliquots, targeted qPCR validation, clinical severity scores, and longitudinal sampling will be needed to clarify the clinical meaning of quantitatively measured HBoV1 levels (Mijač et al., 2024).

Several sequence-level findings supported the HBoV1 assignment. Dominant remapping signals, HBoV1 reference-genome coverage, and partial VP1/NS1 phylogenetic placement all pointed to HBoV1 within the analyzed regions. HBoV1 reads were not limited to a short local region, and sample-level coverage metrics were summarized to make the sequencing evidence more transparent. At the same time, genome coverage alone cannot exclude laboratory contamination, index hopping, or sample cross-contamination (Jurasz et al., 2021). The laboratory environmental surface controls analyzed in this study did not support HBoV1 contamination from the tested laboratory surfaces, but these controls were not equivalent to extraction blanks, no-template controls, or library blanks. This limitation should be considered when interpreting low-level mNGS signals.

The phylogenetic and recombination analyses also require cautious interpretation. The study-derived VP1 and NS1 fragments clustered within the HBoV1 clade, supporting genotype assignment within the analyzed regions. VP1 is a structural protein involved in viral particle formation and host interaction, whereas NS1 is a non-structural protein involved in viral replication (Cotmore et al., 2019; Colazo Salbetti et al., 2023). RDP4 did not detect recombination events involving the study-derived HBoV1 fragments. One candidate event was observed only among HBoV3/HBoV4 reference sequences in the NS1 alignment and did not affect the HBoV1 assignment. Because the available sequences were partial fragments rather than complete genomes or full-length genes, these analyses cannot be used to infer whole-genome diversity or genome-wide recombination. Complete-genome sequencing would be needed to evaluate HBoV1 evolutionary features more comprehensively (Kapoor et al., 2010; Fu et al., 2011).

This study has several limitations. First, the pediatric group was small, with only 30 BALF samples, and the adult and pediatric groups were not balanced. Second, healthy BALF controls were not available, so we could not estimate the background detection rate of HBoV in asymptomatic individuals or fully distinguish active lower respiratory tract infection from incidental carriage (Fry et al., 2007). Third, the study was based on retrospective residual clinical specimens, and clinical information was incomplete. Although **Supplementary Table 1** includes available age, sex, diagnosis, ICU admission status, oxygen requirement, and hospitalization information, ICU stay duration, discharge outcome, and complete admission-to-discharge records were not consistently available. These missing data limited formal clinical correlation between HBoV mNGS signal status, disease severity, hospitalization duration, and outcome. Fourth, independent qPCR validation and standard process controls, including extraction blanks, no-template controls, library blanks, and positive controls, were not included in the original workflow. Fifth, the phylogenetic and recombination analyses were based on partial gene fragments rather than complete genomes.

Despite these limitations, this study provides BALF-based mNGS data for interpreting HBoV1 detection in a selected regional pediatric pneumonia cohort. The findings support HBoV1 as the dominant HBoV genotype detected in these pediatric BALF samples, but they should be interpreted as evidence of viral detection and cohort-level association rather than proof of causative infection. Larger prospective studies with standardized process controls, qPCR validation, longitudinal sampling, complete clinical information, and broader pathogen testing will be needed to clarify when HBoV1 detection reflects active infection, co-detection, prolonged shedding, or incidental viral presence.

## 5 Conclusions

Based on viral metagenomic sequencing of BALF samples, this study described the detection pattern, mNGS-derived abundance, genome coverage, and partial genetic features of HBoV in a retrospective pneumonia cohort. HBoV1 was frequently detected in pediatric BALF samples, whereas no HBoV mNGS signal-positive samples were identified among the adult samples analyzed. All HBoV mNGS signal-positive samples were assigned to HBoV1 as the dominant genotype and showed consistent HBoV1 genome coverage and VP1/NS1 phylogenetic placement. These findings suggest that HBoV1 detection may be relevant to the interpretation of some pediatric BALF samples, but they should be regarded as evidence of viral detection and cohort-level association rather than proof of causative infection. The main contribution of this study lies in providing BALF-based mNGS evidence for HBoV1 detection in a regional pediatric pneumonia cohort, rather than identifying a new respiratory genotype. Larger studies with quantitative validation, longitudinal sampling, more complete clinical information, and multi-pathogen analysis are needed to determine when HBoV1 detection reflects active infection, co-detection, prolonged shedding, or incidental viral presence.

## Data Availability

The raw sequencing data generated in this study have been deposited in the Genome Sequence Archive (GSA) of the National Genomics Data Center (NGDC), China National Center for Bioinformation (CNCB)/Beijing Institute of Genomics, Chinese Academy of Sciences, under BioProject accession PRJCA061470 and GSA accessions CRA040926 and CRA045159. The HBoV1 VP1 and NS1 fragments have been deposited in GenBase under accession numbers C_AA360814.1 to C_AA360832.1. These data are publicly accessible through the NGDC repositories.

## Supplementary material

Supplementary Figure 1 Analysis of HBoV1 whole-genome sequencing depth and coverage in HBoV mNGS signal-positive pediatric samples. The heatmap illustrates the distribution of sequencing depth across the HBoV1 reference genome (DQ000495.1, total length 5,217 bp). For visualization, per-site depth values were summarized into non-overlapping 50-bp genomic bins by calculating the mean depth within each bin. The binned depth values were then transformed using log10(depth + 1), with colors ranging from light yellow to dark brown representing lower to higher depth values.

Supplementary Figure 2 Viral family-level signals detected in laboratory environmental surface control libraries. The heatmap shows read-level DIAMOND/MEGAN viral family-level signals in three laboratory environmental surface controls, including the PCR instrument surface, centrifuge inner surface, and biosafety cabinet inner surface. For visualization, MEGAN normalized values were transformed using log10(value + 1). The detected signals were mainly phage-related or environmental viral taxa.

Supplementary Figure 3 Read-level viral co-detection profile in pediatric BALF samples. The heatmap shows selected human- or vertebrate-associated viral genera detected by read-level DIAMOND/MEGAN screening in pediatric BALF samples. Samples are annotated according to HBoV mNGS signal status, which was defined using the predefined ≥10 total HBoV-mapped read screening threshold. For visualization, MEGAN normalized values were transformed using log10(value + 1). HBoV-related taxa were not included because HBoV was evaluated separately by targeted HBoV1–4 remapping. Bacteriophage-related, environmental, algal, insect-associated, giant DNA virus-related, retrovirus-like, and low-confidence background viral signals were not included in the heatmap.

Supplementary Table 1 Clinical information, sequencing quality metrics, and HBoV read counts for BALF samples included in this study. The table summarizes available sample-level information, including sample accession, BioSample name, patient group, sampling location, clinical diagnosis and co-detected pathogens when available, raw reads, clean reads after trimming, host-depleted reads, FastQC-estimated sequence duplication rate, total HBoV-mapped reads, and genotype-level HBoV read counts. Raw reads, clean reads after trimming, and host-depleted reads are reported as the total number of reads from R1 and R2 combined. Clean read percentage was calculated as clean reads after trimming divided by raw reads, and host-depleted percentage was calculated as host-depleted reads divided by clean reads after trimming. The FastQC-estimated sequence duplication rate was calculated as the mean percent duplicates of R1 and R2 reported by FastQC after trimming. HBoV RPM and genome coverage metrics for HBoV mNGS signal-positive pediatric samples are provided in **Supplementary Table 2**.

Supplementary Table 2 HBoV1 genome coverage metrics for HBoV mNGS signal-positive pediatric samples. Coverage breadth was defined as the percentage of the HBoV1 reference genome covered by at least one read. Mean and median depth values were calculated across the whole HBoV1 reference genome, with uncovered positions included as zero-depth sites.

Supplementary Table 3 Recombination screening results based on RDP4 analysis. Recombination screening was performed using RDP4 based on VP1 and NS1 nucleotide alignments. The RDP, GENECONV, BootScan, MaxChi, Chimaera, SiScan, and 3Seq methods were used with Bonferroni correction and a highest acceptable P value of 0.05. The sequences were treated as linear DNA sequences. No recombination event involving the study sequences was detected. One candidate event was detected among HBoV3/HBoV4 reference sequences in the NS1 alignment, but it did not involve sequences obtained in this study.

Supplementary Table 4 Threshold sensitivity analysis of HBoV mNGS signal detection under alternative total HBoV-mapped read thresholds. This table summarizes the number and frequency of HBoV mNGS signal-positive samples in pediatric and adult groups under different total HBoV-mapped read thresholds. Exact binomial 95% confidence intervals were calculated for detection frequencies. Fisher’s exact test was used to compare pediatric and adult groups at each threshold. Because zero signal-positive samples were observed in the adult group at several thresholds, Haldane–Anscombe correction was used to estimate odds ratios and 95% confidence intervals for effect-size reporting.

Supplementary Table 5 Viral signals and HBoV remapping results in laboratory environmental surface control libraries. Three laboratory environmental surface control libraries were analyzed, including a PCR instrument surface control, a centrifuge inner-surface control, and a biosafety cabinet inner-surface control. HBoV1–4 read counts were obtained by targeted remapping against representative HBoV genotype reference sequences. Viral family-level signals were summarized from read-level DIAMOND/MEGAN screening using MEGAN normalized values. No HBoV1 reads were detected in any of the three environmental controls. Two HBoV3-mapped reads were observed in the centrifuge inner-surface control, which was below the predefined ≥10 total HBoV-mapped read screening threshold and was not accompanied by HBoV1 signal.
